# ATP5J regulates microglial activation via mitochondrial dysfunction, exacerbating neuroinflammation in intracerebral hemorrhage

**DOI:** 10.3389/fimmu.2024.1509370

**Published:** 2024-12-13

**Authors:** Naixin Ren, Hutao Zhang, Tao Li, Huafang Ji, Zhen Zhang, He Wu

**Affiliations:** Department of Pathology, First Clinical Hospital, Harbin Medical University, Harbin, China

**Keywords:** intracerebral hemorrhage, ATP5J, microglia, mitochondrial reprogramming, secondary brain injury

## Abstract

Microglial-mediated neuroinflammation is crucial in the pathophysiological mechanisms of secondary brain injury (SBI) following intracerebral hemorrhage (ICH). Mitochondria are central regulators of inflammation, influencing key pathways such as alternative splicing, and play a critical role in cell differentiation and function. Mitochondrial ATP synthase coupling factor 6 (ATP5J) participates in various pathological processes, such as cell proliferation, migration, and inflammation. However, the role of ATP5J in microglial activation and neuroinflammation post-ICH is poorly understood. This study aimed to investigate the effects of ATP5J on microglial activation and subsequent neuroinflammation in ICH and to elucidate the underlying mechanisms. We observed that ATP5J was upregulated in microglia after ICH. AAV9-mediated ATP5J overexpression worsened neurobehavioral deficits, disrupted the blood-brain barrier, and increased brain water content in ICH mice. Conversely, *ATP5J* knockdown ameliorated these effects. *ATP5J* overexpression also intensified microglial activation, neuronal apoptosis, and inflammatory responses in surrounding tissues post-ICH. ATP5J impaired microglial dynamics and reduced the proliferation and migration of microglia to injury sites. We used oxyhemoglobin (OxyHb) to stimulate BV2 cells and model ICH *in vitro*. Further *in vitro* studies showed that *ATP5J* overexpression enhanced OxyHb-induced microglial functional transformation. Mechanistically, *ATP5J* silencing reversed dynamin-related protein 1 (Drp1) and mitochondrial fission 1 protein (Fis1) upregulation in microglia post-OxyHb induction; reduced mitochondrial overdivision, excessive mitochondrial permeability transition pore opening, and reactive oxygen species production; restored normal mitochondrial ridge morphology; and partially restored mitochondrial respiratory electron transport chain activity. *ATP5J* silencing further alleviated OxyHb-induced mitochondrial dysfunction by regulating mitochondrial metabolism. Our results indicate that ATP5J is a key factor in regulating microglial functional transformation post-ICH by modulating mitochondrial dysfunction and metabolism, thereby positively regulate neuroinflammation. By inhibiting ATP5J, SBI following ICH could be prevented. Therefore, ATP5J could be a candidate for molecular and therapeutic target exploration to alleviate neuroinflammation post-ICH.

## 1 Introduction

Intracerebral hemorrhage (ICH) is a severe cerebrovascular disease that accounts for approximately 15% to 20% of all strokes, with high morbidity and mortality rates (1). ICH-induced brain injury occurs through primary and secondary mechanisms. A primary injury is caused by the acute mass effect of a hematoma, resulting in brain tissue compression and deformation and increased intracranial pressure. A secondary injury is initiated by erythrocyte debris and blood components, leading to inflammatory responses, oxidative stress, neuronal apoptosis, mitochondrial dysfunction, and blood–brain barrier (BBB) disruption (2). Despite remarkable advancements in diagnosis and surgical procedures, treatment options for primary brain injuries remain limited (3). Consequently, most experimental studies focus on the mechanisms of ICH-induced secondary injuries to identify effective therapeutic targets.

Neuroinflammation is a critical host defense response (4–6). Following ICH, a strong inflammatory response contributes substantially to early brain injury (7–9). Microglia, the brain’s immune cells, quickly respond to ICH and are recruited to the bleeding site (10–13). They play a crucial role in early brain injury. Notably, they possess remarkable functional flexibility that allows them to adjust to dynamic inflammatory responses (14, 15). Emerging evidence suggests that microglial function status can be regulated by mitochondrial function and metabolism (16–18). The mitochondria are essential for metabolism and energy production. When the mitochondrial respiratory chain complex is inhibited, mitochondrial dysfunction occurs in ischemic stroke models, leading to increased infarct volume and exacerbated BBB disruption (19). Microglia in Alzheimer’s disease (AD) exhibit an increased Drp1 expression, a decreased Mitofusin expression, excessive mitochondrial fragmentation, and impaired mitochondrial and mtDNA exocytosis (20). These mitochondrial alterations target peripheral astrocytes and activate innate immune pathways, thereby inducing inflammatory responses. This effect creates a positive feedback loop that promotes an inflammatory microenvironment, further activating microglia and exacerbating neuronal death (21). Therefore, mitochondrial imbalance in microglia is vital in their functional transformation and disease progression.

Mitochondria are central to cellular energy metabolism and homeostasis, with ATP synthase serving as the terminal enzyme of the electron transport chain (ETC), catalyzing ATP production through oxidative phosphorylation (22, 23). Beyond its canonical role in ATP synthesis, ATP synthase also maintains mitochondrial membrane stability and regulates cellular bioenergetics. Under pathological conditions, such as ischemia and neurodegeneration, ATP synthase can reverse its activity, hydrolyzing ATP and exacerbating bioenergetic deficits, which contributes to mitochondrial dysfunction (24). Dysregulation of mitochondrial function, including disturbances in metal ion homeostasis and oxidative stress, is strongly associated with inflammation and cellular damage in neurodegenerative diseases (25, 26). While ATP synthase’s overall function is well established, the contributions of individual subunits to pathological processes remain poorly understood. Among these subunits, ATP5J, also known as ATP synthase membrane subunit F6, has been implicated in mitochondrial dynamics and cellular stress responses. Proteomic and transcriptomic studies suggest that ATP5J may influence mitochondrial function and contribute to neuroinflammatory pathways, particularly in glial cells, including microglia (27, 28). However, its role in intracerebral hemorrhage (ICH), a condition marked by neuroinflammation and secondary mitochondrial damage, remains unexplored.

This study investigated the effects of ATP5J on the inflammatory response and neurological outcomes of ICH to explore its potential mechanisms. We found that *ATP5J* overexpression significantly exacerbated hematoma, edema, and BBB damage following ICH, whereas *ATP5J* knockdown improved functional recovery. We also showed that ATP5J influenced microglial function through reactive oxygen species (ROS) accumulation and mitochondrial metabolic reprogramming, thus regulating the inflammatory response after ICH. These findings suggested that ATP5J could serve as an effective molecular target for studying the mechanisms of ICH-induced SBI.

## 2 Materials and methods

### 2.1 Ethical declaration

All animal procedures were performed in strict compliance with the guidelines for the care and use of laboratory animals and approved by the Ethical Committee on Animal Experiments of Harbin Medical University (Approval No.2021138). Twelve-week-old male C57BL/6 mice were housed in cages under the following conditions: controlled temperature and humidity, 12 h light/dark cycle, and free access to food and water.

### 2.2 Animals and models

Twelve-month-old male C57BL/6 mice, weighing 25–28 grams (Changsheng Company, Liaoning Province, China), were housed in an environment with temperatures of 18–26°C and humidity levels of 40%–70%. Noise levels were maintained below 85 dB, and ammonia concentrations were kept below 20 ppm. The mice were ventilated 8–12 times per hour and monitored under a standard 12 h light/dark cycle. Food and water were provided ad libitum. Efforts were made to optimize the animal experimentation strategy by providing humane care and adhering to the “3R” principles (reduce, replace, refine) in animal experiments through the reduction of the number of animals used and the suffering associated with experimental procedures. Deep anesthesia followed by cervical dislocation was applied in accordance with euthanasia guidelines.

The C57BL/6 mice were randomly divided into six groups: sham operated, ICH-24h, ICH-72h, ICH-7d, ICH-14d, and ICH-28d. Additionally, three groups of six mice each were formed: ICH + vehicle, ICH + ATP5J KD, and ICH + ATP5J OE. An *in vivo* ICH model was established by injecting type IV collagenase into the striatum (29, 30). The mice were fasted for 12 h before surgery, and water was withheld 4 h before surgery. After the mice were adequately anesthetized with isoflurane, they were placed in a prone position on a stereotaxic apparatus. The skin on the scalp was disinfected, and a T-shaped incision was made to expose the skull. In the skull, an injection site measuring 3 mm × 1 mm × 5 mm was created, and a hole was drilled. The striatal region of the mice was located using the stereotaxic apparatus (2.2 mm to the left and 1.0 mm forward). The solution was then slowly injected into the region. After a 5 min interval, the microinjector was carefully withdrawn. The drilled hole was closed with bone wax, and the incision site was sutured. After the operation, the mice were kept in a ventilated room and allowed to recover on a heating blanket at 37°C. In the ICH group, 0.5 μL of collagenase (4 U)/heparin (2 U) in 1 μL of sterile saline was injected; in the sham-operated group, an equal volume of saline solution was introduced into the left striatal region.

### 2.3 Adeno−associated virus 9

Holes were drilled in the skull, and 5 µL of AAV containing either *ATP5J* or the vector (Gikai Bio, China) was injected into the left striatum at a rate of 0.4 µL/min by using a micro-syringe. The injection site was 2.2 mm lateral to the bregma, 1.0 mm anterior, and 2.7 mm deep. The titer of the ATP5J-KD and ATP5J-OE lentivirus (Gikai Bio, China) was 1E+12 TU/ml. Transfection was achieved via *in situ* injection into the left striatum 4 weeks before ICH was induced, which was confirmed via western blot analysis.

### 2.4 Real-time (quantitative) reverse transcription -polymerase chain reaction

RNA was extracted from the brain tissue or cells by using Trizol reagent (Sigma-Aldrich, St. Louis, MO) and reverse-transcribed to cDNA (TaKaRa, Shiga, Japan). qRT-PCR was then performed using a Thermo Fisher PCR machine (Thermo Fisher Scientific, Waltham, MA), and the primers (**Supplementary Table 1**) were synthesized by Shanghai Sangong Biotechnology Co. Data were analyzed as previously described. Each experiment was repeated thrice, and all reactions were performed in triplicate.

### 2.5 Western blotting

The proteins from the tissue and cells surrounding the hematoma were extracted using protein lysate (Beyotime, Jiangsu, China) and quantified with a BCA protein assay kit (Beyotime, China). Equal amounts of the proteins were separated using standard sodium dodecyl sulfate-polyacrylamide gel electrophoresis (Beyotime, China) and transferred to poly- vinylidene difluoride (PVDF) membranes (Thermo Fisher Scientific, USA) of varying pore sizes based on their molecular weight. The membranes were washed thrice with TBST (Tween-20: TBS = 1:1000) for 5 min per wash. They were then blocked with 5% skimmed milk powder for 1 h at 37°C and incubated with primary antibodies (**Supplementary Table 2**) at 4°C overnight. They were washed again and incubated with horseradish peroxidase-conjugated secondary antibodies (either goat anti-rabbit or anti-mouse, Proteintech, China) for 1.5 h at 37°C. The next day, the membranes were observed, and their gray values were measured using an enhanced chemiluminescence substrate (7 Sea biotech, China) and ImageJ software (version 1.4.3.67). GAPDH, actin, or β-tubulin (Proteintech, China) was used as an internal reference band. All experimental procedures were repeated thrice.

### 2.6 Hematoma volume

The mice were anesthetized and euthanized. Then, their brain tissue was dissected and fixed following perfusion. The brain tissue was serially sectioned in the coronal plane by using a stainless steel sectioning mold, and each section was 1 mm thick. The sections were placed on 1 cm grid paper and photographed. The area of the hemorrhagic foci per section was multiplied by 1 mm and the number of sections to calculate the ICH volume of each section by using ImageJ (10).

### 2.7 Neurological scores and neurobehavioral function test

After ICH, the mice experienced motor and sensory impairments. Their neurobehavioral function was assessed 3 days after ICH via the Modified Neurological Severity Score (MNSS) (31), the Cornering Test (CT) (32), and the Neurobehavioral Function Test (NFT) by two researchers who were blinded to the experimental grouping (33, 34). In the NFT, a 24-point MNSS scale was used. Scores were obtained by observing the movement of the side of the ICH and the contralateral forelimb of the awake mice. Additionally, the body symmetry, turning direction, gait, 45° slope climbing, and forced turning of the mice were evaluated to objectively assess the degree of neurological deficits. The tests were conducted by observers who were unaware of the grouping and study context. Each group was given a score of 0–24 points; the higher the score, the more severe the nerve damage. During the cornering test, the mice were directed toward a 30°corner, and they exited by turning either right or left. The test environment was quiet and protected from light, and at least 30 s of rest was provided between trials. The percentage of correct results out of 10 trials was calculated.

### 2.8 Forelimb placement test

The forelimb placement test was conducted to evaluate sensorimotor function and recovery following intracerebral hemorrhage (ICH). Mice were gently held in an upright position with their hindlimbs supported, allowing the forelimbs to move freely. The contralateral forelimb relative to the lesion site was gently tapped against the edge of a table to elicit a reaching response. Each mouse underwent 10 trials per forelimb, and the number of successful placements was recorded. A correct placement was defined as the mouse extending its forelimb and accurately placing the paw on the table edge. Failed attempts included absent, incomplete, or inaccurate paw movements. To ensure consistency, the test was conducted in a controlled environment with minimal external distractions. The results were expressed as the percentage of successful placements per 10 trials for each forelimb. Group comparisons were performed using appropriate statistical analyses to assess differences in motor recovery.

### 2.9 Accelerated rotarod test

The accelerated rotarod test was conducted to assess motor coordination and balance in mice. Testing was performed using a rotarod apparatus with a rod diameter of 3 cm. The rod was programmed to accelerate from 4 revolutions per minute (rpm) to 40 rpm over a 300-second period. Mice were acclimatized to the apparatus by performing three preliminary trials one day before testing, with each trial lasting 2 minutes at a constant speed of 4 rpm. On the test day, each mouse underwent three trials with a 30-minute inter-trial interval to avoid fatigue. The latency to fall, defined as the time in seconds from the start of rotation until the mouse fell off the rod or gripped the rod for two consecutive revolutions, was recorded for each trial. The average latency across the three trials was calculated for each mouse. Mice that failed to remain on the rod for at least 10 seconds during the first trial were excluded from subsequent analysis to ensure consistency. Data were analyzed using one-way ANOVA followed by Bonferroni corrections for multiple comparisons, with significance set at p < 0.05.

### 2.10 Brain water content

The brain edema index was evaluated using the wet and dry weight technique as outlined in a previous study (12). Sodium pentobarbital was administered intraperitoneally 72 h after ICH was induced, and the entire brain was excised. The brain was bisected along the midline, and each hemisphere was promptly weighed to record its wet weight. Then, it was dehydrated for 72 h at 100°C, and its dry weight was measured. The brain water content (BWC) was calculated using the following formula: (wet tissue weight − dry tissue weight)/wet tissue weight × 100%.

### 2.11 Immunohistochemistry

The paraffin sections were prepared and dewaxed with water before antigens were retrieved. Once cooled, they were evenly coated with 3% BSA and left at 37°C for 30 min. They were gradually added with primary antibodies and incubated flat in a humidified chamber at 4°C overnight. They were then washed thrice with PBS and incubated with the corresponding secondary antibody at 37°C for 40 min.DAB chromogenic solution was added dropwise and the staining was observed under a microscope and terminated at any time. Next, the sections were re-stained with hematoxylin for approximately 3 min, washed with water, differentiated with a hydrochloric acid alcohol differentiation solution for a few seconds, and washed again. Lastly, their blue hue was recovered using ammonia, and the sections were rinsed with running water. After the sample was dehydrated and sealed in neutral resin, the sections were observed under a microscope, and images were collected for data analysis.

### 2.12 Immunofluorescence

After being fixed with 4% neutral paraformaldehyde for 24 h, the brains were progressively dehydrated using 20%, 30%, and 40% sucrose solutions until the brain tissues settled at the bottom of the container. The brain tissues were embedded in OCT freezing embedding gel, and pure cryosections were cut at a thickness of 15 µm by using a cryostat. The resulting sections were air-dried at ambient temperature and then baked at 65°C for 30 min. After removal the frozen sections, antigens were retrieved on the frozen sections by using sodium citrate. After the retrieval solution was washed out with PBS, the sections were blocked with a 5% BSA solution for 1 h at room temperature. They were then incubated with the primary antibody targeting the desired protein at 4°C overnight. They were washed with PBST and incubated with the corresponding secondary antibody at 37°C in the dark for 1.5 h. The sections were washed again with PBST and incubated with DAPI for 30 min. Lastly, the sections were mounted, observed under a fluorescence microscope, photographed, and analyzed (10). For ICH mice, ROIs were specifically selected from the peri-hematoma region on the ipsilateral (injury) side of the brain, encompassing tissue directly adjacent to the hematoma core. For Sham mice, equivalent regions were selected from the corresponding anatomical location in the striatum on the ipsilateral side.

Fluorescence images were captured using a fluorescence microscope under consistent exposure settings. To minimize variability, three non-overlapping fields were randomly selected from each ROI for imaging. DAPI-positive nuclei and ATP5J fluorescence signals were quantified using ImageJ software with identical thresholding applied across all samples.

### 2.13 Terminal deoxynucleotidyl transferase dUTP Nick−end labeling (TUNEL) staining

A TUNEL assay was conducted to evaluate apoptosis after ICH (15) in cells and paraffin sections by using a TUNEL assay kit (Beyotimeh, China) in accordance with the manufacturer’s instructions. Briefly, dewaxed and dehydrated brain paraffin sections or treated cells were exposed to the TUNEL reaction solution at 37°C for 1 h. After being washed with PBS, the slides were incubated with DAPI for 20 min and sealed with an anti-fluorescence quenching sealer. The number of TUNEL-positive cells was observed and counted via fluorescence microscopy. Immunofluorescence images were captured using a confocal fluorescence microscope at 40× magnification. Representative images were selected from the peri-hematoma region in ICH mice, where TUNEL-positive cells were most abundant. For quantitative analysis, three non-overlapping fields were randomly selected from the peri-hematoma area. The number of TUNEL-positive cells was counted using ImageJ software.

### 2.14 Transmission electron microscopy

Cells from the OxyHb+NC, OxyHb+Vector, OxyHb+ATP5J-KD, and OxyHb+ATP5J-OE groups were collected and processed into 100 nm sections. These sections were stained with 4% uranyl acetate for 20 min and 0.5% lead citrate for 20 min. They were visualized and quantified via transmission electron microscopy (TEM) using a Philips Tecnai 10 microscope.

### 2.15 ROS

Brain ROS levels were measured as an oxidative stress indicator by using a ROS assay kit (Beyotimeh, China) and a flow cytometry method. Tissue and cell samples were harvested, homogenized, and centrifuged at 12,000 × *g* and 4°C for 10 min. Then, the supernatant was collected, and ROS levels were identified using the oxidant-sensitive probe 2,7-dichlorofluorescein diacetate. The fluorescence signals of 10,000 cells were detected within 30 min by using an Agilent 2070R flow cytometer to obtain a curve. All FCS data were analyzed using FlowJo 10.8.1.

### 2.16 Cell culture and transfection

BV2 cells were cultured in high-glucose DMEM containing 10% FBS, 1% penicillin, and 1% streptomycin at 37°C with 5% CO_2_. They were treated with 20 μM oxyhemoglobin for 24 h or left untreated as a blank control to mimic the *in vitro* ICH model (35). For *ATP5J* knockdown experiments, the cells were transfected with Lipofectamine 8000 (Thermo Fisher Scientific, USA) by using the constructed interference control (OxyHb+NC) and small interfering RNA (siRNA ATP5J). The Coding sequences of ATP5J was inserted into a plasmid containing the Cytomegalovirus promoter, named ATP5J-OE, with its control named Vector. The cells were incubated at 37°C for 24 h. Afterward, the medium was changed, and 20 μM OxyHb was added to continue incubation for another 24–48 h. The proteins were extracted or used for other assays. The experiment was divided into the following groups: Control, OxyHb+NC, OxyHb+Vector, OxyHb+ATP5J-KD, and OxyHb+ATP5J-OE.

### 2.17 Mitochondrial permeability transition Pore measurement

mPTP was assessed using a mitochondrial membrane potential (TMRE) assay kit (Beyotime, China). TMRE was used to label active mitochondria, and mPTP was determined on the basis of the fluorescence intensity of the mitochondrion-isolated TMRE. BV2 cells were inoculated in six-well plates and incubated with 400 nmol/L TMRE at 37°C away from light for 1 h. After two brief washes with warm 1× PBS (37°C), the cells were observed under a fluorescence microscope, and the number of positive cells was counted.

### 2.18 ATP content

The ATP content in the cells was measured using an ATP content assay kit (Beyotime, China) in accordance with the provided instructions. First, cell homogenates were prepared for the ATP assay. The cells were mixed with 10 times their volume of the extract and homogenized on ice. Then, the supernatant was collected after centrifugation at 12,000 × *g* for 10 min. The absorbance values of each reaction mixture were measured using a spectrophotometer at 340 nm.

### 2.19 Cell counting kit-8

Cell viability was evaluated using the CCK-8. The cells were cultured in 96-well plates at a volume of 100 μL per well and treated with various supernatant groups for 24 h. Subsequently, 10 μL of CCK-8 reagent was added to each well, and the plates were further incubated at 37°C for 1 h. The number of viable cells was assessed by recording the absorbance at 450 nm. Control cells with 100% viability (CCK-8) were included for comparison to normalize the relative assessment of microglial damage.

### 2.20 Transwell assay

Cell migration was assessed using Costar Transwell plates (0.4 μm pore size, Corning, China). The microglial density was 1×10^3^ cells. Then, 200 μL of the serum-free medium was added to the upper chamber, while 500 μL of the complete medium with 10% fetal bovine serum was added to the lower chamber. After 24 h of incubation, the microglia were fixed to the underside of the insert in the presence or absence of a damage-inducing factor (20 μM OxyHb + HT22 cells) by using 90% ethanol for 15 min. Subsequently, they were stained with 0.1% crystal violet for 20 min. The experiment was repeated thrice. The migrated cells were counted and observed under an inverted microscope with an objective lens. At least three distinct fields of view were chosen, and the average number of the migrated cells in these fields was calculated.

### 2.21 Determination of lactic acid content

Cells were divided into groups, and samples were taken and diluted from each group. The enzyme working solution was prepared using a lactate test kit (Solarbio, China) in accordance with the manufacturer’s instructions. The respective samples were added to a blank tube, a standard tube, and an assay tube and then mixed well. After 10 min of reaction in a water bath at 37°C, 2 mL of the termination solution was added to each tube. The absorbance of each well was detected at 530 nm by using an enzyme marker; the blank tube was used to adjust to zero. The absorbance of each tube was then determined, and the lactic acid content of each tube was calculated using the formula provided in the kit.

### 2.22 ETC complex enzyme activity assay

After the cell samples were pretreated according to the experimental groups, cell supernatants were collected through centrifugation. The protein concentration of some supernatants was determined, while the remaining supernatants were used to assess the enzyme activity in accordance with the instructions of the respective assay kits (Solarbio, China).

### 2.23 Statistical analysis

Data were statistically analyzed using GraphPad Prism 8 and expressed as mean ± standard deviation. Unpaired t-tests were used to analyze statistical differences between two groups, and one- or two-way ANOVA was performed to examine differences between multiple groups. Statistical significance was set at p < 0.05.

## 3 Results

### 3.1 ATP5J expression increases predominantly in microglia and exhibits a time-dependent effect following ICH

**Figure 1:**
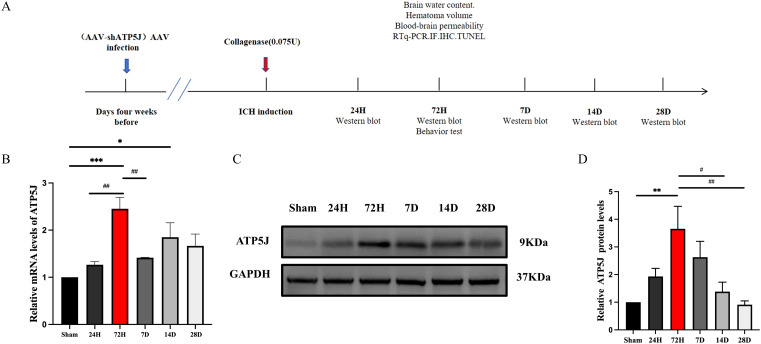
Expression profile of ATP5J in ICH mice. **(A)** Flow chart illustrating the experimental design *in vivo*. Assays were conducted at various time points post-ICH onset. **(B)** mRNA levels of *ATP5J* in perihematomal tissues at the indicated time points. **(C)** Protein levels of ATP5J in perihematomal tissues at the indicated time points. **(D)** Quantification of the ATP5J protein expression in perihematomal tissues at the indicated time points. Statistical significance: *p < 0.05, **p < 0.01, ***p < 0.001 compared with Sham; ^#^ *p* < 0.05, ^##^ *p* < 0.01 compared with 72H. Data were presented as mean ± SE, where the mean values of the Sham group were normalized to 1. Group sizes: n = 6 per group.

**Figure 2:**
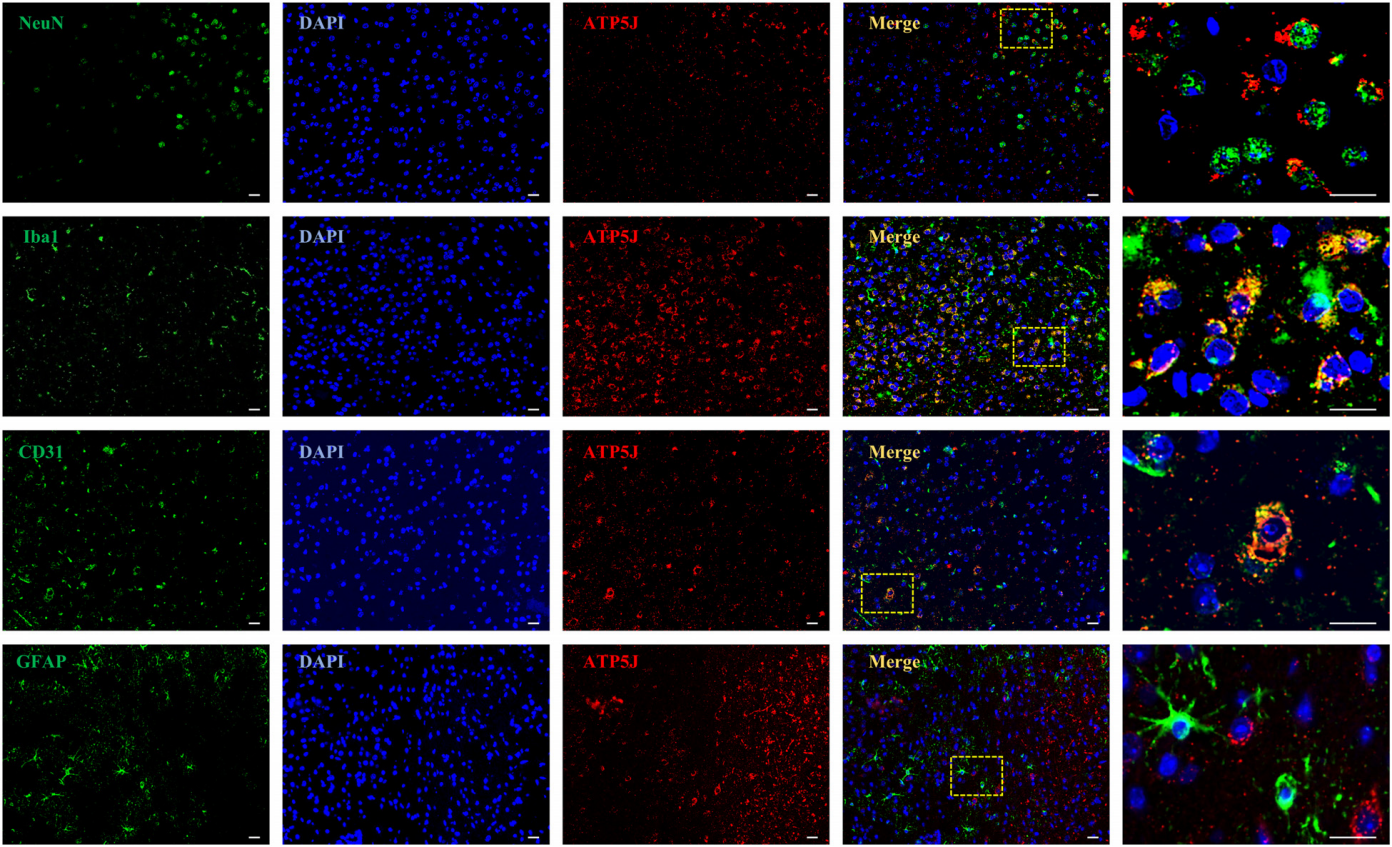
Spatial localization of ATP5J in ICH mice. The following assays were performed after ICH at 72H. Double immunofluorescent analysis was performed using ATP5J antibodies (red) and the neuronal marker NeuN (green), the microglial marker Iba-1 (green), the vascular marker CD31, or the astrocyte marker (green) in brain sections. Nuclei were fluorescently labeled with 4,6-diamino-2-phenylindole (DAPI; blue; n = 6). Representative images of the ICH (72H) groups are shown. Images at 20× magnification were used for structural localization, while 40× mirror magnification highlights finer details. Scale bar = 20 μm.

To investigate the temporal and spatial expression of ATP5J after ICH, we analyzed the brain tissues from the control and type IV collagenase-induced ICH groups of C57BL/6 mice via western blot and double immunofluorescence staining. Western blot analysis showed that ATP5J expression was low under normal conditions. After ICH, ATP5J protein levels significantly increased at 24 h, peaked at 72 h, gradually decreased, and returned to baseline levels on day 14 (**Figures 1C, D**). The mRNA level of *ATP5J* also peaked 72 h after ICH (**Figure 1B**). Double immunofluorescence revealed that the ATP5J expression in neuronal cells, microglia, astrocytes, and vascular endothelial cells was low under normal conditions (**Figure 2**). However, following ICH, the number of ATP5J-positive cells around the hematoma significantly increased, and the most pronounced elevation was observed in microglia. Similarly, the ATP5J level in OxyHb-induced BV2 cells *in vitro* for 24 h was high. (**Supplementary Figure 3**) These data suggested that ICH enhanced the ATP5J expression in microglia in a time-dependent manner.

### 3.2 ATP5J worsens neurological deficits and brain damage in mice after ICH

**Figure 3:**
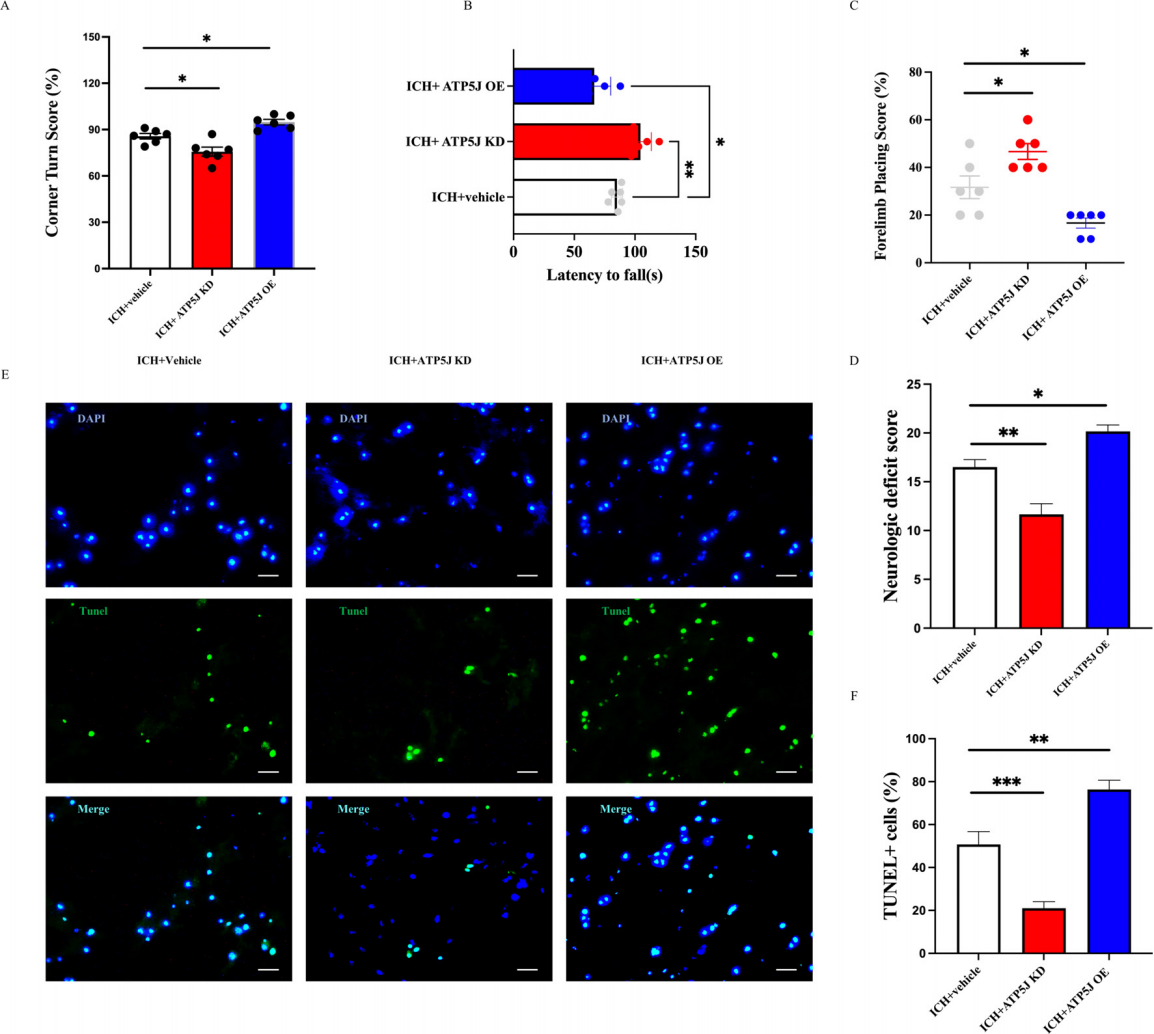
ATP5J modulates behavioral outcomes, neurological deficits, and cell apoptosis following intracerebral hemorrhage (ICH). **(A)** *ATP5J* knockdown significantly reduces corner turn scores, indicating improved motor function, while *ATP5J* overexpression worsens performance. **(B)** *ATP5J* knockdown extends the latency to fall, suggesting enhanced motor coordination, whereas *ATP5J* overexpression shortens the latency. **(C)** *ATP5J* knockdown improves forelimb placing scores, reflecting enhanced motor function, while *ATP5J* overexpression leads to a decline. **(D)** ATP5J knockdown markedly lowers neurological deficit scores, indicating functional recovery, while ATP5J overexpression exacerbates deficits. **(E)** *ATP5J* knockdown decreases the number of TUNEL-positive cells, indicating reduced apoptosis, whereas *ATP5J* overexpression significantly increases apoptotic cell counts. Representative images were captured at 40× magnification, and the scale bar = 20 μm (n = 6 per group). **(F)** Quantification of TUNEL-positive cells. Statistical significance: *p < 0.05, **p < 0.01, ***p < 0.001.

To investigate the effect of ATP5J on ICH, we modified the ATP5J protein levels through knockdown and overexpression by topically administering adeno-associated viral vectors to the striatum of C57BL/6 mice. ICH modeling was performed by injecting type IV collagenase *in situ* 4 weeks after viral administration. Various behavioral tests were conducted 72 h after ICH to evaluate the influence of ATP5J expression levels on neurological deficits. A flowchart of the process of the *in vivo* experiment is shown in **Figure 1A**. We found that the mice with ICH exhibited deteriorated neurological function scores (**Figure 3E**) compared with those of the control mice, decreased correct placement in the forelimb placement test (**Figure 3C**), and an abnormally increased rate of escaping to the left in the cornering test (**Figure 3A**). These results indicated a repetitive-like phenotype. Notably, the mortality rate of the mice in the ICH group overexpressing ATP5J (ICH+ATP5J OE group) was significantly higher than that of the littermate control mice. Neurological scores were significantly lower in the ICH group than in the littermate controls. However, the mice in the ICH+ATP5J KD group showed milder injury-induced behavioral deficits in neurological function scores, forelimb placement tests, and cornering tests (**Figure 3D**). Furthermore, the results from the accelerated rod-turning assay indicated that the motor, sensory, and balance abilities of the mice with ICH with knocked down ATP5J improved (**Figure 3B**). These findings suggested that *ATP5J* overexpression could worsen ICH-induced neurobehavioral deficits, while *ATP5J* knockdown could mitigate brain injury. In summary, after ICH, ATP5J exacerbated hemorrhage-induced neurological deficits.

To investigate the effect of ATP5J on cell apoptosis around the hematoma core, we performed TUNEL staining to assess the total number of apoptotic cells in the perihematomal region. Our findings indicated that ICH significantly increased the number of TUNEL-positive cells in the perihematomal region on the hemorrhagic side (**Figures 3D, F**). The number of TUNEL-positive cells in the ATP5J-overexpressing ICH mice was significantly higher than that in the control group, indicating an exacerbation of apoptosis. Conversely, ATP5J knockdown at 72 h significantly reduced the number of TUNEL-positive cells. Overall, these findings indicate that ATP5J overexpression exacerbates cell apoptosis, thereby worsening neurological outcomes following ICH, whereas ATP5J knockdown mitigates apoptotic activity, potentially facilitating neural recovery.

### 3.3 ATP5J worsens cerebral edema and BBB integrity post-ICH

**Figure 4:**
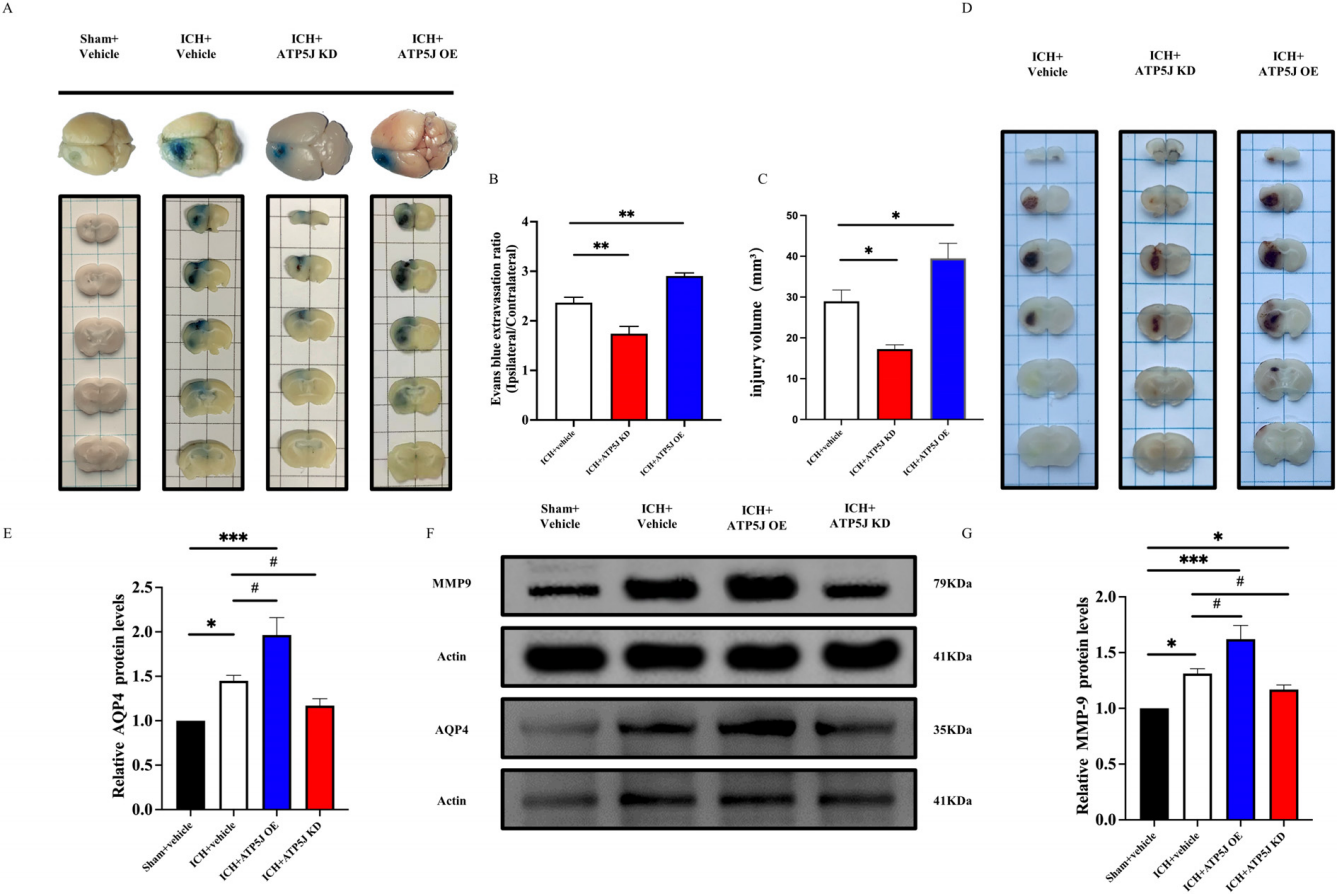
Effects of the knockdown and overexpression of *ATP5J* on ICH-induced hematoma expansion, brain edema, and blood–brain barrier permeability. **(A)** Representative images of Evans blue extravasation. **(B)** Quantification of Evans blue extravasation 72 h after ICH (n = 6 per group). **(C, D)** Representative images of the expansion size of the hematoma and the quantification of the hemorrhagic volume in different groups. **(G)** Quantification of brain water content 72 h after ICH (n = 6 per group). **(E-G)** AQP4 and MMP-9 protein levels in mouse brain tissues around the hematoma 72 h after ICH. Statistical significance: *p < 0.05, **p < 0.01, ***p < 0.001; ^#^ *p* < 0.05, n = 6 per group.

After ICH, secondary brain damage occurs because of hematoma formation and perihematomal edema (2). The hemorrhagic lesion volume was measured 72 h after ICH. The hematoma volume, identified as deposition in the striatal region on the hemorrhagic side, peaked at 72 h. The mice in the ICH+ATP5J OE group exhibited an increased hematoma size and worsened perihematomal edema in the cortex and basal ganglia. Conversely, the mice in the ICH+ATP5J KD group showed a transition from bright red oxygenated hematomas to dark-brown deoxygenated hematomas or methemoglobin; their hematoma size and ipsilateral cerebral water content significantly decreased at 72 h (**Figures 4C, D, G**). Since BBB damage is a critical factor in ICH-induced SBI (3), we assessed the effect of ATP5J on the BBB integrity and permeability post-ICH. Evans blue extravasation assay (**Figures 4A, B**) showed that Evans blue leakage in the ICH+ATP5J OE group significantly increased compared with those in the sham and ICH groups. The Evans blue concentration in the striatum on the hemorrhagic side was lower in the ICH+ATP5J KD group than in the ICH group. Furthermore, the expression levels of matrix metalloproteinase-9 (MMP-9) and aquaporin-4 (AQP4) were significantly lower in the ICH+ATP5J KD group than in the ICH group (**Figures 4E-G**). Therefore, ATP5J significantly worsened ICH-induced hematoma and edema, consequently compromising the BBB integrity.

### 3.4 ATP5J reduces microglial branching and enhances the microglial activation status in the peri-hemorrhagic region

**Figure 5:**
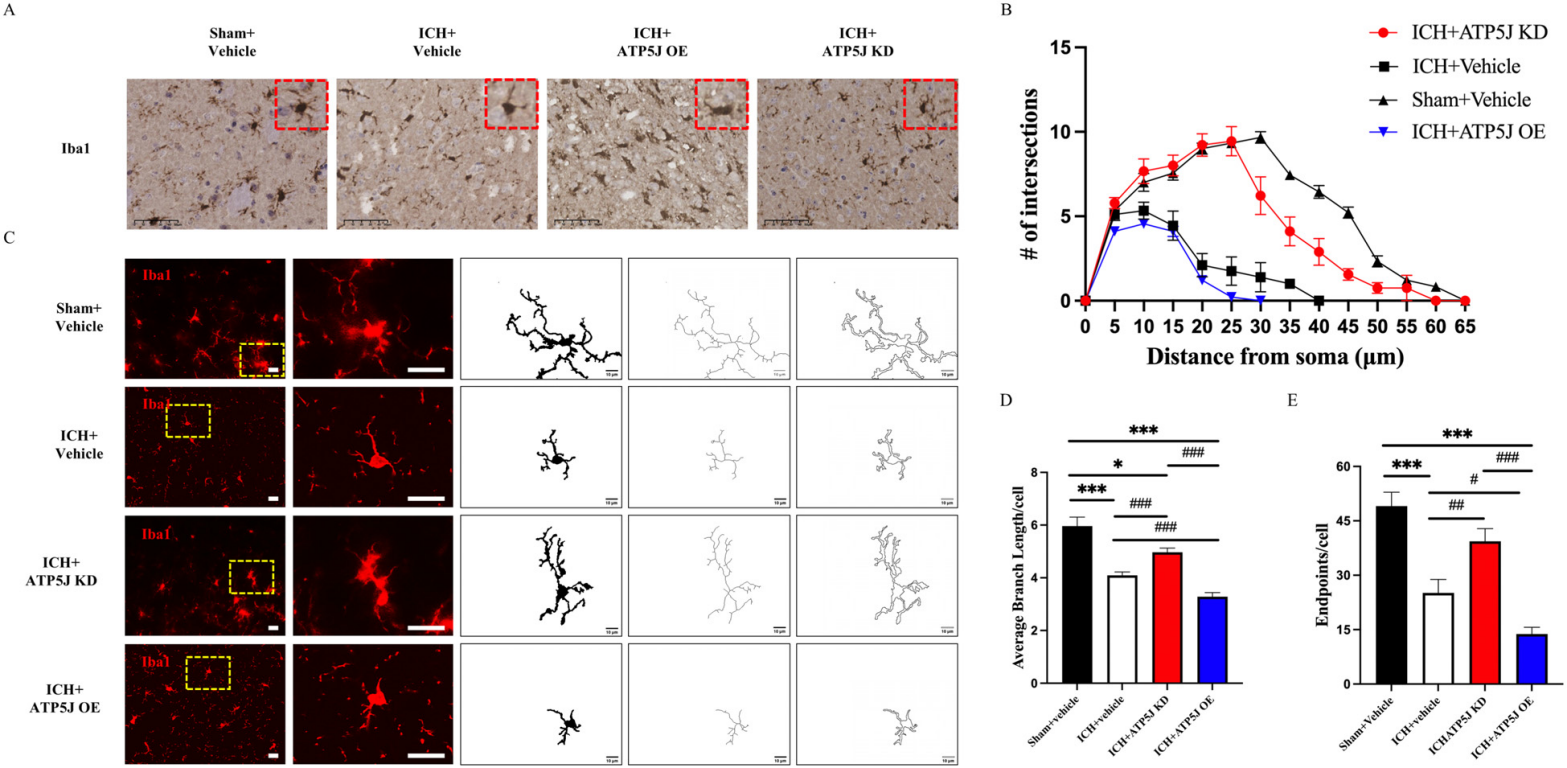
Reducing effects of ATP5J knockdown on microglial activation and morphological changes after ICH. **(A)** Immunohistochemistry staining of activated microglia (Iba-1) in the perihematomal region, 72 hours after ICH. Scale bar = 50 μm (n = 6 per group). **(B)** Representative images of activated microglia (red) in the perihematomal region, used for the measurement of morphological parameters and quantitative analysis, including intersections **(C)**, average branch length **(D)**, and endpoint branch numbers **(E)**. Scale bar = 20 μm (n = 6 per group). Statistical significance: *p < 0.05, ***p < 0.001; ^#^p < 0.05, ^##^p < 0.01, ^###^p < 0.001.

To investigate the mechanisms of ATP5J-mediated neuroprotection after ICH, we examined the effects of *ATP5J* knockdown and overexpression on microglial function in the perihematomal region. Morphological changes in the microglia were assessed using immunofluorescence and Iba1 immunohistochemical staining to evaluate microglial activation. Immunofluorescence staining revealed a significant enhancement in the activation state of microglia, characterized by enlarged soma and reduced branching complexity, around the injury site in the ATP5J-overexpressing group compared with that in the ICH group (**Figure 5A**). Additionally, the activated microglia displayed a larger cytosol and reduced branching, indicating that their activation was enhanced (**Figures 5B–E**). Conversely, *ATP5J* knockdown partially restored the homeostatic morphology of the microglia.

**Figure 6:**
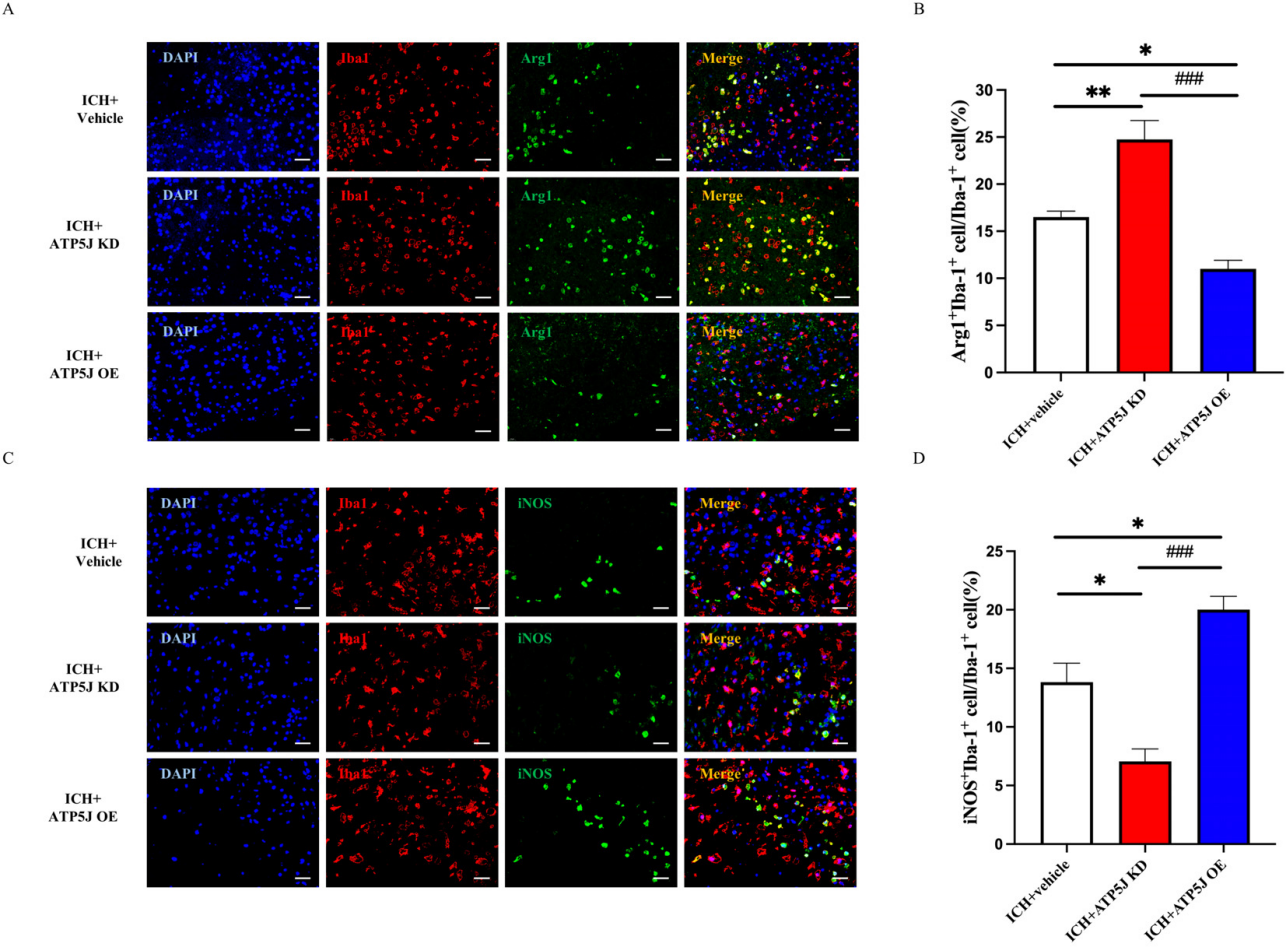
Attenuating effects of ATP5J knockdown on the pro-inflammatory state of microglia *in vivo*. **(A, C)** Representative images of double immunofluorescent analysis using Iba-1 antibodies (red) and either the pro-inflammatory marker iNOS (green) or the anti-inflammatory marker Arg-1 (green) in brain sections from the perihematomal region, 72 hours after ICH. Scale bar = 20 μm (n = 6 per group). **(B, D)** Quantification of Arg-1+Iba-1+ cells and iNOS+Iba-1+ cells in the perihematomal region. Statistical significance: *p < 0.05, **p < 0.01, ^###^p < 0.001.

**Figure 7:**
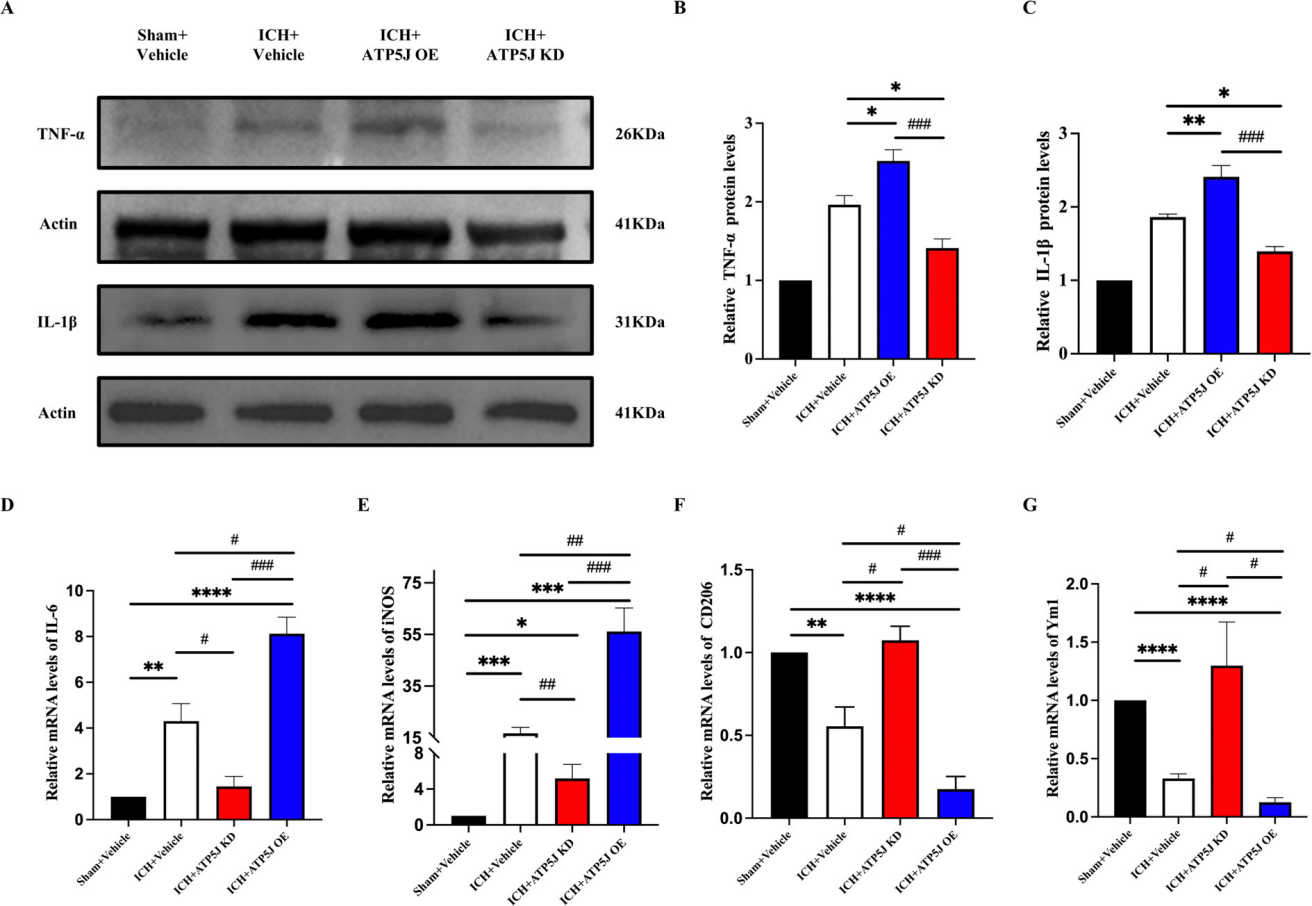
Increased brain inflammation by *ATP5J* overexpression and reduced inflammatory response by *ATP5J* knockdown. **(A–C)** Immunoblot analysis of TNF-α and IL-1β expression in the perihemorrhagic tissues of ICH mice. **(D, G)** mRNA expression levels of inflammatory markers (*IL-6* and *iNOS*) in ICH mice after *ATP5J* knockdown or overexpression. **(E, F)** mRNA expression levels of anti-inflammatory markers (*CD206* and *Ym-1*) in ICH mice after *ATP5J* knockdown or overexpression. Statistical significance: *p < 0.05, **p < 0.01, ***p < 0.001; ****p < 0.0001, ^#^ *p* < 0.05, ^##^ *p* < 0.01, ^###^ *p* < 0.001. n = 6 per group.

Microglia, which rapidly activate and switch subtypes in response to acute brain injury, secrete pro-inflammatory or anti-inflammatory cytokines that influence ICH outcomes (14, 15). In the present study, immunofluorescence staining demonstrated that the *ATP5J* overexpression increased Iba1+/iNOS+ microglia and decreased Iba1+/Arg1+ microglia around the hematoma 72 h after ICH compared with those in the ICH+Vehicle group (**Figure 6**). Western blot analysis showed that the TNF-α and IL-1β expression levels in the ipsilateral hemisphere significantly decreased after ATP5J knockdown compared with those detected 72 h after ICH (**Figures 7A–C**). qRT-PCR analysis revealed that the levels of inflammatory markers (*IL-6* and *iNOS*) increased (**Figures 7D, E**) and the levels of anti-inflammatory markers (*CD206* and *Ym-1*) decreased in the ICH+ATP5J OE group compared with those in the controls (**Figures 7F, G**). Conversely, the inflammatory marker levels of the ICH+ATP5J KD group were significantly lower than ICH+Vehicle group and its anti-inflammatory marker levels were higher than ICH+Vehicle group. These findings suggested that ATP5J regulated microglial-induced neuroinflammation and provided cerebroprotection following ICH.

### 3.5 ATP5J regulates the dynamics of microglia

**Figure 8:**
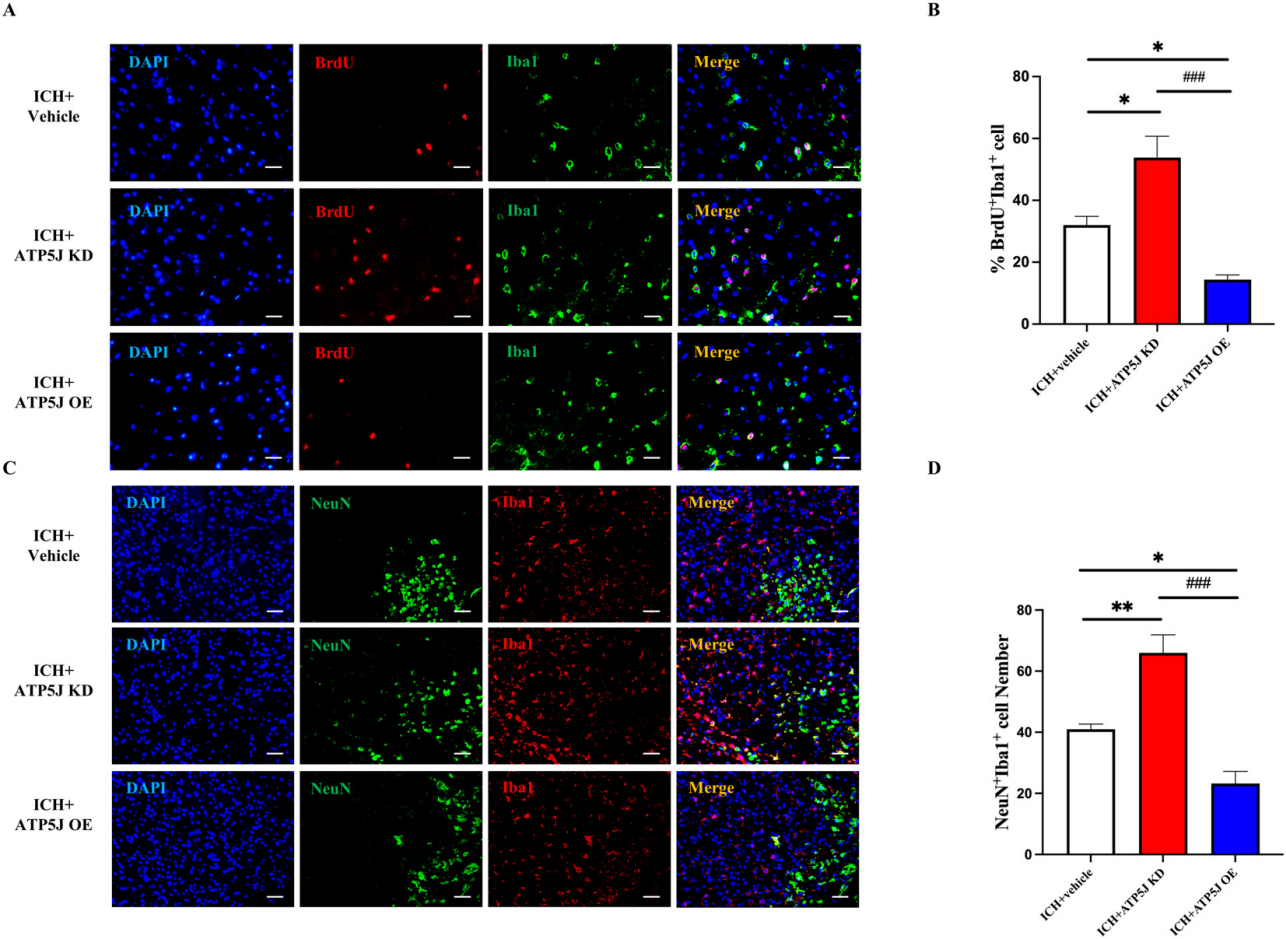
Reduction in the migration and repopulation of microglia to the perihematomal region via ATP5J expression upregulation after ICH *in vivo*. **(A, B)** Representative images and quantification of the immunofluorescence staining of BrdU and Iba-1 in the perihematomal tissues of ICH mice in each group. Scale bar = 20 μm (n = 6 per group). **(C, D)** Aggregation of Iba-1+ cells around neurons in brain tissues on the hemorrhagic side of ICH mice. Scale bar = 20 μm (n = 6 per group). Statistical significance: *p < 0.05, **p < 0.01, ^###^ *p* < 0.001.

To assess the effect of ATP5J on microglial proliferation, we used BrdU labeling with Iba1. The number of BrdU+/Iba1+ microglia was significantly reduced in the ICH+ATP5J OE group and increased in the ICH+ATP5J KD group compared with that in the ICH+Vehicle group (**Figures 8A, B**). Immunofluorescence double-labeling revealed that the number of Iba1+ cells around the injured neurons in the peri-hemorrhagic region decreased *in vivo*, but they were restored by *ATP5J* knockdown (**Figures 8C, D**). These findings indicated that the *ATP5J* overexpression reduced microglial proliferation after hemorrhage, while *ATP5J* knockdown increased microglial cells in the perihematomal region.

**Figure 9:**
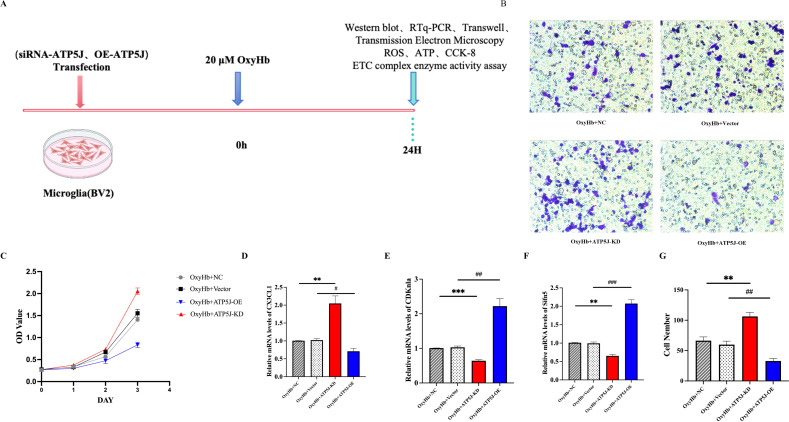
Reduction in the migration and repopulation of microglia to the perihematomal region via ATP5J expression upregulation after ICH *in vitro*. **(A)** Flow chart showing the experimental design *in vitro*. **(B, G)** Transwell assay was performed to determine the migratory capacity of BV2 cells after *ATP5J* knockdown or overexpression. **(C)** CCK-8 assay showed that the number of BV2 cells significantly decreased after *ATP5J* overexpression *in vitro*; this effect was mitigated by *ATP5J* knockdown. **(D–F)** mRNA expression levels of the BV2 cell homeostasis marker *CX3CL1*, the cell cycle arrest inducer *CDKn1a*, and the DNA replication inhibitor *slfn5* after *ATP5J* knockdown or overexpression. Statistical significance: **p < 0.01, ***p < 0.001; *p* < 0.05, ^#^ *p* < 0.05, ^##^ *p* < 0.01, ^###^ *p* < 0.001. n = 3 per group.

Then, we conducted *in vitro* experiments on BV2 cells exposed to 20 μM OxyHb for 24 h (**Figure 9A**). *In vitro* migration assays showed that the migrating microglia in the OxyHb+ATP5J-OE group significantly decreased. Conversely, the microglial presence around the injury site in the OxyHb+ATP5J-KD group increased (**Figure 9B**). *In vitro* CCK-8 assays confirmed that *ATP5J* knockdown accelerated microglial proliferation (**Figure 9C**). qRT-PCR analysis showed that the expression of the microglial homeostasis marker *CX3CL1* in the knockdown group increased, while the mRNA levels of *CDKN1A* and *slfn5*, which are markers of cell cycle arrest and DNA replication inhibition, decreased (**Figures 9D-G**). Therefore, a high ATP5J expression following hemorrhage likely impaired microglial dynamics, while *ATP5J* knockdown possibly enhanced microglial proliferation and migration.

### 3.6 ATP5J exacerbates mitochondrial dysfunction in microglia after ICH by regulating metabolism and ROS accumulation

**Figure 10:**
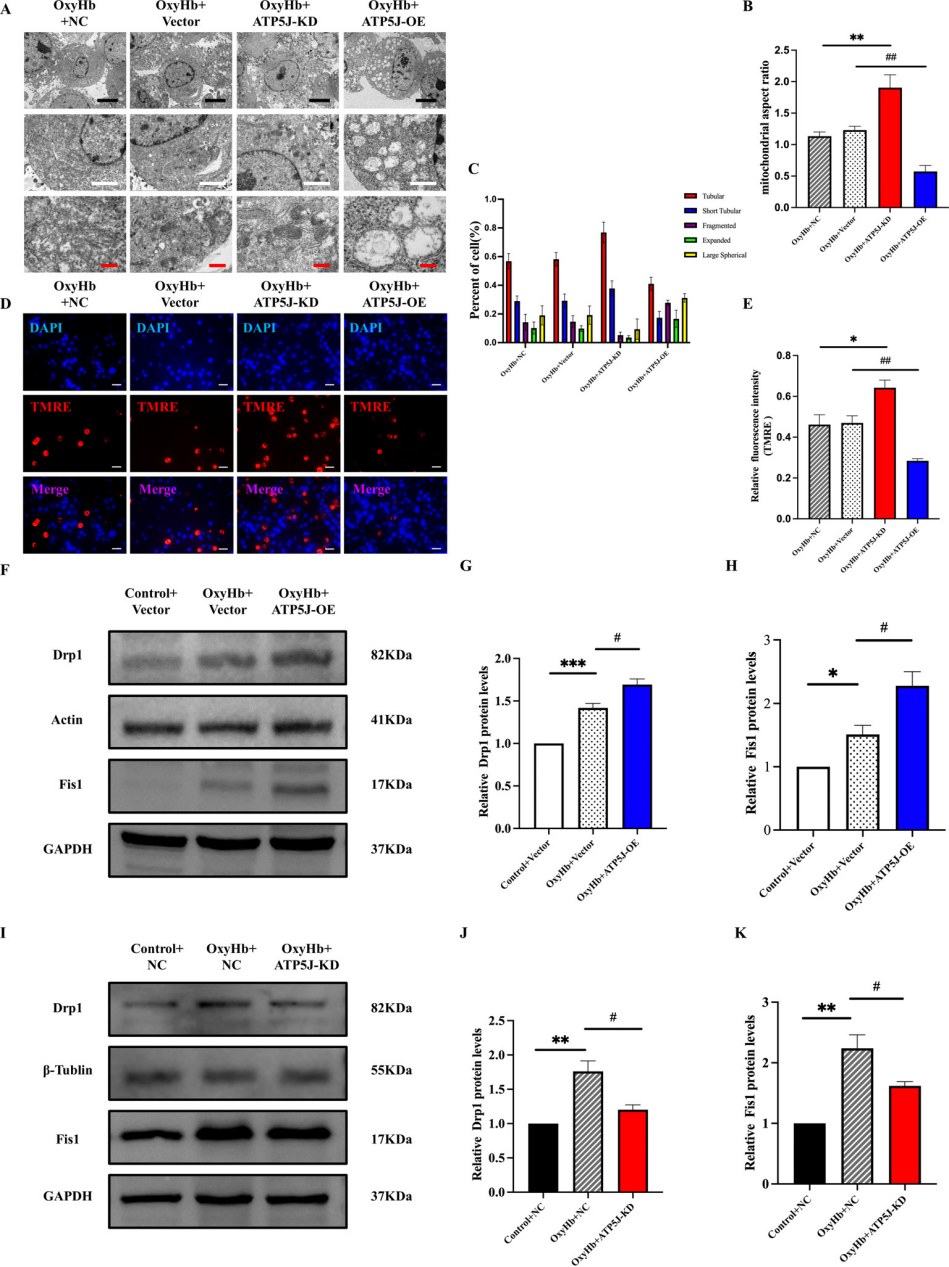
Attenuation of the OxyHb-induced mitochondrial structure and dysfunction of BV2 cells via *ATP5J* knockdown. **(A)** Representative images of mitochondrial morphology under different conditions: the OxyHb+NC and OxyHb+Vector groups showed inner membrane collapse; the OxyHb+ATP5J-KD group exhibited a decrease in mitochondrial damage; the OxyHb+ATP5J-OE group had an increase in mitochondrial damage. Scale bars: 5 μm (black), 2 μm (white), and 500 nm (red). **(B)** Statistical analysis of the mitochondrial aspect ratio in BV2 cells treated with different methods. **(C)** Statistical analysis of the proportion of different crista structures in BV2 cells: tubular crista (**p < 0.01, ###*p* < 0.001), short tubular crista (*p < 0.05, ##*p* < 0.01), fragmented crista (**p < 0.01, ###*p* < 0.001), expanded crista (*p < 0.05, #*p* < 0.05), and large spherical crista (ns, *p* < 0.05). **(D, E)** Mitochondrial membrane potential measured via TMRE staining. Aggregated red fluorescence denotes normal mitochondria, while decreased red fluorescence indicates damaged mitochondria and decreased ΔΨm. Scale bar = 20 μm. **(F, I)** Protein expression levels of the mitochondrial division proteins Drp1 and Fis1 in each group. **(G, H, J, K)** Quantification of Drp1 and Fis1 protein expression in each group. “OxyHb+NC” represents BV2 cells transfected with NC siRNA and treated with 20 μM OxyHb; “OxyHb+Vector” includes cells transfected with an empty vector and treated with 20 μM OxyHb; “Control+NC” includes cells transfected with NC siRNA without OxyHb treatment. Statistical significance: *p < 0.05, **p < 0.01, ***p < 0.001; ^#^ *p* < 0.05, ^##^ *p* < 0.01, ^###^ *p* < 0.001. n = 3 per group.

Transmission electron microscopy revealed the ultrastructure of microglial mitochondria. In the control group, intact globular and elongated tubular mitochondrial structures with curved cristae were observed. Conversely, in the post-ICH samples, crista protrusions were lost, the crista membrane collapsed, and the mitochondrial size was reduced. The mitochondrial fragmentation in the OxyHb+ATP5J OE group was more severe, and the aspect ratio was elevated; however, these effects were reversed by *ATP5J* knockdown (**Figures 10A-C**).

Western blot analysis revealed that the expression levels of Drp1 and Fis1 (20, 21), key proteins in mitochondrial fission, in the OxyHb+ATP5J OE group were significantly higher than those in the OxyHb+Vector group (**Figures 10F-K**). TMRE staining detected changes in mitochondrial membrane potential (ΔΨm). Healthy mitochondria exhibit a high negative internal charge, causing TMRE to aggregate in the mitochondrial matrix and emit bright-orange fluorescence. Because of cell damage, mitochondrial membrane potential loss occurs, and TMRE is released into the cytoplasm, thereby reducing fluorescence intensity (22, 23). In **Figures 10D, E**, the orange fluorescence intensity in the OxyHb+Vector and OxyHb+ATP5J OE groups decreased compared with that in the control group, indicating the increased mPTP opening and loss of membrane potential. This effect was reversed in the OxyHb+ATP5J KD group.

**Figure 11:**
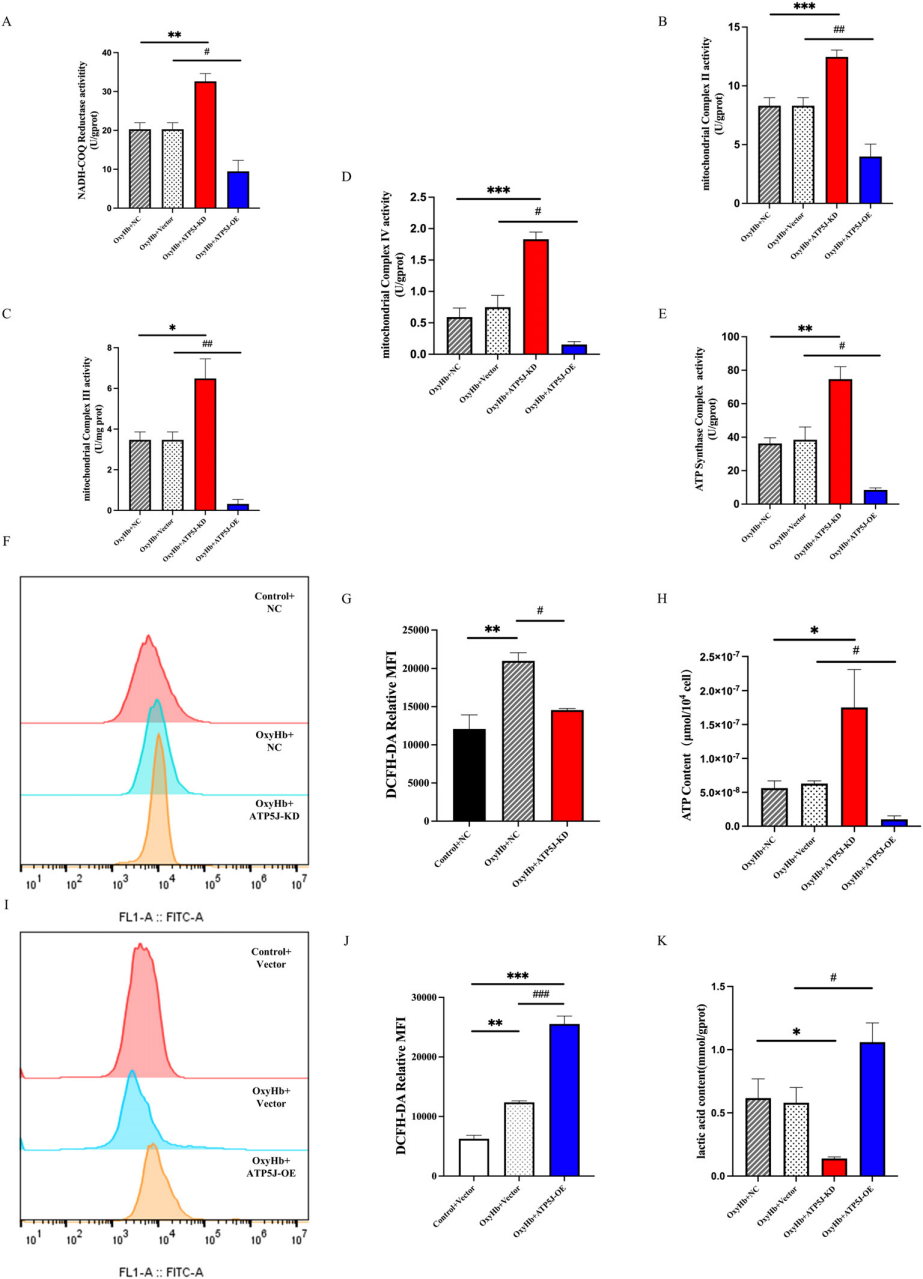
Stimulation of mitochondrial metabolic reprogramming and ROS accumulation in BV2 cells by the OxyHb-induced upregulation of ATP5J expression. **(A-E)** Measurement of the enzymatic activities of mitochondrial electron transport chain complexes. **(F, I, G, J)** Flow cytometry analysis of intracellular ROS levels in BV2 cells from different groups. **(H)** Changes in ATP contents in BV2 cells from different groups. **(K)** Changes in lactate contents in BV2 cells from different groups. “OxyHb+NC” represents BV2 cells transfected with NC siRNA and treated with 20 μM OxyHb; “OxyHb+Vector” includes cells transfected with an empty vector and treated with 20 μM OxyHb; “Control+NC” includes cells transfected with NC siRNA without OxyHb treatment. Statistical significance: *p < 0.05, **p < 0.01, ***p < 0.001; ^#^ *p* < 0.05, ^##^ *p* < 0.01, ^###^ *p* < 0.001. n = 3 per group.

Lactate plays a critical role in cellular energy metabolism. Excess lactate uptake and metabolism within cells result in elevated production of ROS, which disrupt normal mitochondrial energy processes (36–38). Regulating mitochondrial metabolism is crucial for microglial phenotypic transition under neuroinflammatory conditions such as ICH. In the OxyHb+NC and OxyHb+Vector groups, the ROS levels significantly increased after mitochondrial damage, which was mitigated by *ATP5J* knockdown (**Figures 11F, I, G, J**). Mitochondrial respiratory function was assessed by measuring the enzyme activities of oxidative respiratory chain complexes. The assay results (**Figures 11A-E**) showed that the enzyme activities of oxidative respiratory chain complexes I–V were reduced in the OxyHb+NC and OxyHb+Vector groups; these activities were further significantly reduced after *ATP5J* overexpression. Conversely, *ATP5J* knockdown partially restored the activities of complexes I and III likely because of their role in releasing superoxide toward the mitochondrial matrix. This finding indicated that post-ICH, mitochondrial respiration was significantly impaired. Thus, the impairment was further exacerbated by *ATP5J* overexpression but was alleviated by *ATP5J* knockdown.

Intracellular ATP levels showed that *ATP5J* knockdown in the OxyHb+ATP5J-KD group restored the ICH-induced ATP reduction while decreasing the glycolytic byproduct lactate concentrations compared with those in the OxyHb+NC groups (**Figures 11H, K**). In summary, our results demonstrated that a high ATP5J expression exacerbated mitochondrial structural and functional dysfunction after ICH. Conversely, *ATP5J* knockdown offered neuroprotection by regulating mitochondrial ROS accumulation and metabolic shifts.

## 4 Discussion

In this study, we discovered a novel role of ATP5J in modulating neuroinflammation and neuronal damage after ICH. Our data showed that ATP5J upregulation in the damaged striatal regions co-localized with microglial cells and considerably influenced their inflammatory response and function. *ATP5J* overexpression in microglia increased the brain water content and BBB permeability post-ICH, whereas *ATP5J* knockdown improved neurological outcomes and reduced brain damage. These effects were mediated by the ability of ATP5J to promote mitochondrial fragmentation and its associated metabolic reprogramming. Additionally, these findings align with previous studies, which demonstrated that mitochondrial dysfunction exacerbates neuroinflammation by increasing ROS production and disrupting metabolic balance.

The protective effects of *ATP5J* knockdown on microglia are particularly noteworthy. Previous studies highlighted the role of mitochondrial function in microglial activation and neuroinflammation (39). For example, mitochondrial integrity should be maintained to prevent excessive inflammatory responses in microglia (40–42). In our study, *ATP5J* knockdown preserved mitochondrial integrity, reduced ROS production, and enhanced mitochondrial respiration and ATP production; thus, the pro-inflammatory state of microglia was mitigated, and a homeostatic phenotype was promoted.

Neuroinflammation is a major pathological process following ICH, substantially contributing to brain damage and neurological deficits (43, 44). Our findings are consisted with earlier research demonstrating that mitochondrial dysfunction can exacerbate neuroinflammation (45, 46). By regulating mitochondrial fragmentation, ATP5J modulates microglial activation and inflammatory responses. ATP5J inhibition decreases the production of pro-inflammatory cytokines, such as TNF-α and IL-1β, and increases microglial proliferation and migration. These findings suggested a shift toward a protective, anti-inflammatory phenotype.

Our study also showed that *ATP5J* overexpression exacerbated BBB disruption and brain edema, which are critical factors in the progression of ICH-induced injury. The levels of MMP-9 and AQP4, which are known to contribute to BBB permeability and intracerebral edema (47–50), increased. Therefore, *ATP5J* overexpression might disrupt the BBB integrity by promoting MMP-9 production and perturbing AQP4 polarity; consequently, brain edema and injury were exacerbated.

ROS are key inducers of secondary injury after ICH, and they play an essential role in activating pro-inflammatory signaling pathways and neuronal death (51–53). To reveal the regulatory role of ATP5J in microglia, we performed *in vitro* studies and found that *ATP5J* overexpression led to the collapse and vacuolization of mitochondrial cristae, whereas *ATP5J* knockdown alleviated these effects. Mitochondrial membrane potential (MMP), tested via TMRM examination in BV2 cells, showed that *ATP5J* overexpression aggravated the MMP loss, accompanied by ATP reduction and ROS overaccumulation. Conversely, *ATP5J* knockdown recovered the MMP, reduced the ROS levels, and increased the ATP levels (54, 55). These structural changes were associated with functional improvements, including enhanced mitochondrial respiration and reduced ROS levels. Therefore, mitochondrial respiration was significantly impaired post-ICH. This impairment was further exacerbated by *ATP5J* overexpression but was alleviated by *ATP5J* knockdown.

Studies have shown that ATP synthase subunits are involved in regulating intracellular alternative splicing processes (56). Mitochondrial dysfunction, through redox signaling, can modulate alternative splicing and influence inflammatory responses (57, 58). In our study, *ATP5J* knockdown reduced ROS production, preserved mitochondrial integrity, and improved respiration, which likely stabilized splicing processes critical for regulating microglial function. Conversely, *ATP5J* overexpression elevated ROS levels, disrupting splicing regulators and promoting the production of pro-inflammatory cytokines such as TNF-α and IL-1β. These findings suggest that ATP5J-driven mitochondrial dysfunction and ROS overaccumulation may regulate specific splicing events, contributing to microglial activation and exacerbating neuroinflammation after ICH. Targeting ATP5J-mediated mitochondrial function regulation could represent a novel therapeutic strategy for modulating inflammatory responses in neuroinflammatory and other inflammation-driven diseases.

This study primarily highlights ATP5J’s role in ICH, but its involvement in mitochondrial dysfunction and oxidative stress suggests broader relevance to other neurological conditions, such as ischemic stroke and traumatic brain injury. Similar to ICH, mitochondrial fragmentation mediated by Drp1 is critical for neuroinflammation in ischemic stroke, and ATP5J may exert comparable effects by regulating mitochondrial dynamics and oxidative stress. Further validation of ATP5J’s function in additional brain injury models will clarify its broader therapeutic potential.

At the molecular level, ATP5J likely modulates mitochondrial dynamics and redox balance. Evidence indicates that ATP5J influences Drp1 activation and recruitment to mitochondria, essential steps in mitochondrial fission. ATP5J may regulate Drp1 Acetylation or promote the assembly of fission complexes, driving excessive mitochondrial fragmentation and ROS production. These disruptions result in mitochondrial membrane potential loss, impaired ATP synthesis, and oxidative damage. Consistently, our study demonstrated that ATP5J overexpression disrupted cristae structure and aggravated ROS accumulation, while ATP5J knockdown preserved mitochondrial integrity, reduced ROS levels, and enhanced ATP production. Future studies should investigate pathways such as Drp1 Acetylation (59) and ATP5J interactions with fission machinery to elucidate these mechanisms.

ATP5J also demonstrates strong translational potential as a therapeutic target. Modulating ATP5J expression could mitigate oxidative stress, restore mitochondrial function, and reduce neuroinflammation during both the acute and subacute phases of ICH. Emerging RNA interference technologies, such as siRNA and antisense oligonucleotides (ASOs), offer precise downregulation of ATP5J, while small-molecule inhibitors paired with advanced targeted delivery systems may enhance therapeutic specificity and efficacy. These strategies merit further preclinical and clinical exploration to establish their safety and effectiveness, not only in ICH but also in other neuroinflammatory conditions.

Despite these promising findings, our study has several limitations. First, ATP5J expression and function were investigated only in the striatal region of the hemorrhagic hemisphere. Expression levels may vary across brain regions, potentially interacting with different glial cells or neurons. Secondary inflammatory responses or distinct metabolic adaptations, as suggested by prior studies, may occur in areas such as the cortex and hippocampus, underscoring the importance of region-specific analyses. Second, this study focused on a single time point (72 hours post-ICH), corresponding to peak inflammation and mitochondrial dysfunction. Expanding the investigation to earlier and later time points could provide a more comprehensive view of ATP5J’s temporal dynamics. Third, while TUNEL analysis identified apoptotic activity, the absence of neuronal marker co-staining limits our ability to identify specific apoptotic cell types. Future studies should incorporate TUNEL with NeuN or other cell-specific markers to refine these findings.

Additionally, our use of a non-cell-specific AAV system introduces potential off-target effects. Although immunofluorescence analysis showed that ATP5J is predominantly expressed in microglia within the perihematomal region, as evidenced by co-localization with the microglial marker Iba1, transfection of other cell types, such as neurons or astrocytes, cannot be ruled out. This lack of cell specificity complicates the interpretation of results, as ATP5J may have distinct roles depending on the cell type. For instance, ATP5J in microglia may primarily regulate inflammatory responses and phagocytic activity, whereas in neurons it may influence mitochondrial function and synaptic plasticity, and in astrocytes, it may modulate energy metabolism and neurovascular coupling. Dynamic interactions among these cell types further complicate the interpretation of ATP5J’s role in the perihematomal region.

This limitation highlights the need for future studies to employ cell-specific AAV systems or Cre-loxP strategies for precise ATP5J manipulation in specific cell populations. Such approaches could elucidate ATP5J’s unique functional roles in different cell types, providing a more nuanced understanding of its role in neuropathology.

Nevertheless, our findings provide valuable insights into ATP5J’s role in microglia and its contribution to regulating the inflammatory environment in the perihematomal region. Future research should expand the investigation of ATP5J’s roles in other cell types and explore synergistic interactions among different cell populations to better understand its mechanisms in the context of brain injury.

## 5 Conclusions

Our study elucidates that ATP5J upregulation following ICH affects microglial function and inflammatory responses by altering mitochondrial functions and metabolic pathways. ATP5J downregulation in microglia mitigates SBI post-ICH by reducing excessive mitochondrial fission, decreasing ROS production, and enhancing ATP production. Therefore, microglial mitochondrial function and metabolism play a pivotal role in ICH-induced inflammation, and ATP5J may serve as a therapeutic target for anti-inflammatory strategies in ICH.

## Data Availability

The raw data supporting the conclusions of this article will be made available by the authors, without undue reservation.
