# Supplementary material for: ATP5J regulates microglial activation via mitochondrial dysfunction, exacerbating neuroinflammation in intracerebral hemorrhage
Source: Front Immunol. 2024 Dec 13;15:1509370. doi: 10.3389/fimmu.2024.1509370 (PMC11671693; doi:10.3389/fimmu.2024.1509370)
Supplement: Supplementary file 1 [file DataSheet1.pdf]

Supplementary table 1 Antibodies and Sources

Name	Sources
ATP5J	ThermoFisher, USA
MMP-9	Abcam, UK
AQP4	Cell signalling technology, USA
TNF-α	Abcam, UK
IL-1β	Cell signalling technology, USA
Drp1	Abcam, UK
Fis1	Abcam, UK
GAPDH	Affinity, USA
Actin	Affinity, USA
β-tubulin	Affinity, USA

Supplementary table 2 The primers used for RT-qPCR detection.

Gene		Sequences (5′–3′)
ATP5J	Forward	CGCTTCTGTGCTGACCCGAA
	Reverse	GTCACATTGTCCACCCGCTTC
IL-6	Forward	CTTGGGACTGATGCTGGTGACA
	Reverse	GCCTCCGACTTGTGAAGTGGTA
iNOS	Forward	TTTGCTTCCATGCTAATGCGAAAG
	Reverse	GCTCTGTTGAGGTCTAAAGGCTCCG
Ym-1	Forward	CAGGGTAATGAGTGGGTTGG
	Reverse	CACGGCACCTCCTAAATTGT
CD206	Forward	ACAAAGGGACGTTTCGGTG
	Reverse	TGGACATTTGGGTTCAGGAG
CX3CL1	Forward	CGCTTCTGTGCTGACCCGAA
	Reverse	GTCACATTGTCCACCCGCTTC
CDKnla	Forward	AATCCTGGTGATGTCCGACCT
	Reverse	CAATCACGGCGCAACTGCT
Slfn5	Forward	GAAGATGCCTGTCTACCAC
	Reverse	GATTGCACAGACATACCCAT
GAPDH	Forward	AGGTCGGTGTGAACGGATTTG
	Reverse	GGGGTCGTTGATGGCAACA

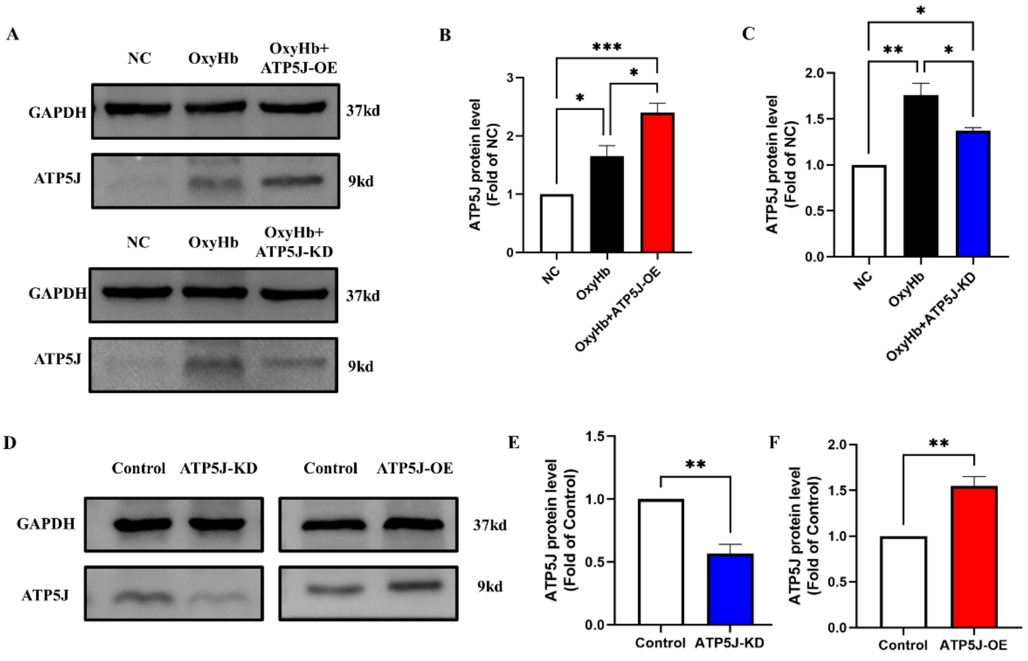

Supplementary Figure 3

Protein validation of ATP5J overexpression and knockdown efficiency in vitro. (A, B, C) Protein validation of ATP5J overexpression and knockdown efficiency in vivo. (D, E, F) Statistical significance: *p < 0.05, **p < 0.01, ***p < 0.001; #p < 0.05, ##p < 0.01, ###p < 0.001. n = 6 per group.

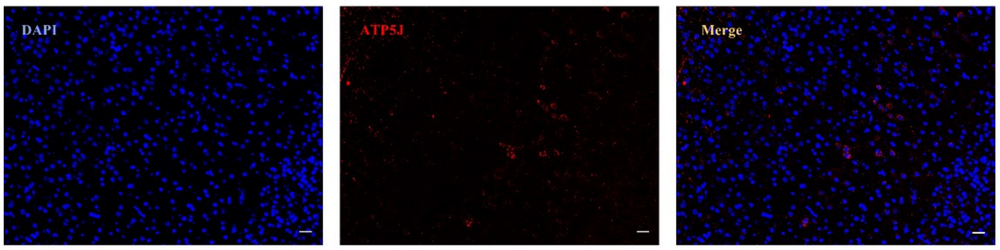

Supplementary Figure 4

The ATP5J level in the Sham mouse. Scale bar = 20 μm.

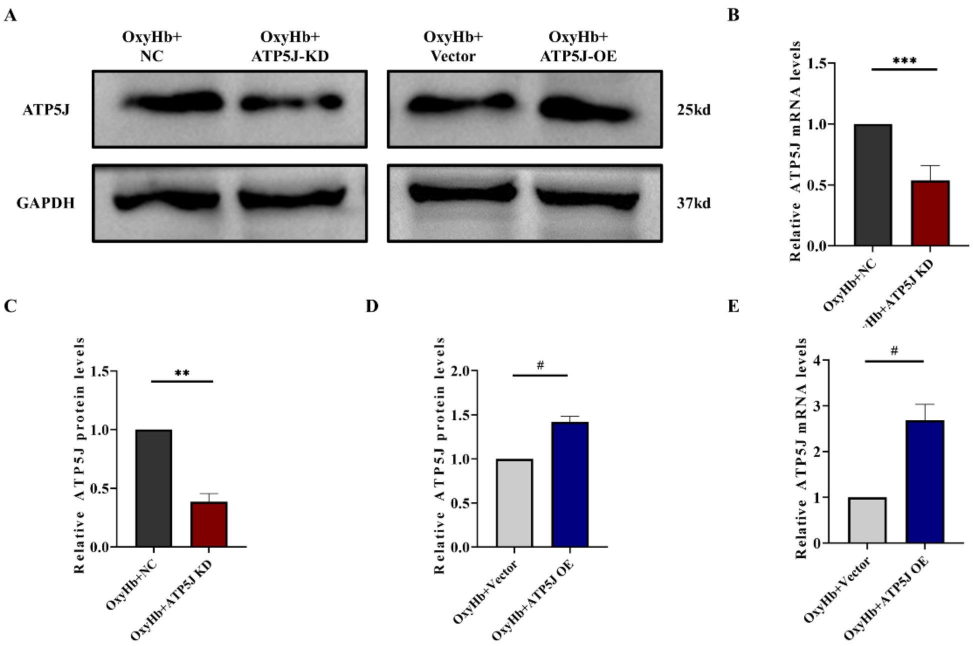

Supplementary Figure 4

(A) Protein validation of ATP5J overexpression and knockdown efficiency in vitro. (B, D) Protein quantification plot of ATP5J overexpression and knockdown efficiency in vitro. (C, E) PCR verification of ATP5J overexpression and knockdown efficiency in vitro. Statistical significance: *p < 0.05, **p < 0.01, ***p < 0.001; #p < 0.05, ##p < 0.01, ###p < 0.001. n = 3 per group.
